# Investigation of the Mechanical and Liquid Absorption Properties of a Rice Straw-Based Composite for Ayurvedic Treatment Tables

**DOI:** 10.3390/ma15020606

**Published:** 2022-01-14

**Authors:** Abhishek Sadananda Madival, Deepak Doreswamy, Shripathi Adiga Handady, Krishna Raghava Hebbar, Shobha Karabylu Lakshminarayana

**Affiliations:** 1 Department of Mechanical and Manufacturing Engineering, Manipal Institute of Technology, Manipal Academy of Higher Education, Manipal 576104, India; abhishek.madival@learner.manipal.edu; 2 Department of Mechatronics Engineering, Manipal Institute of Technology, Manipal Academy of Higher Education, Manipal 576104, India; 3 Division of Ayurveda, Center for Integrative Medicine and Research, Manipal Academy of Higher Education, Manipal 576104, India; shripathi.adiga@manipal.edu (S.A.H.); kr.hebbar@manipal.edu (K.R.H.); 4 Department of Microbiology, Melaka Manipal Medical College, Manipal Academy of Higher Education, Manipal 576104, India; shobha.kl@manipal.edu

**Keywords:** natural composite, rice straw, ayurvedic treatment table, microbial study, tensile strength

## Abstract

Managing rice crop stubble is one of the major challenges witnessed in the agricultural sector. This work attempts to investigate the physical, mechanical, and liquid absorption properties of rice straw (RS)-reinforced polymer composite for assessing its suitability to use as an ayurvedic treatment table. This material is expected to be an alternative for wooden-based ayurvedic treatment tables. The results showed that the addition of rice straw particles (RS_p_) up to 60% volume in epoxy reduced the density of the composite material by 46.20% and the hardness by 15.69%. The maximum tensile and flexural strength of the RS_p_ composite was 17.53 MPa and 43.23 MPa, respectively. The scanning electron microscopy (SEM) analysis showed deposits of silica in the form of phytoliths in various size and shapes on the outer surface of RS. The study also revealed that the water absorption rate (WA) was less than 7.8% for the test samples with 45% volume of RS_p_. Interestingly the test samples showed greater resistance to the absorption of Kottakal Dhanvantaram Thailam (<2%). In addition, the developed samples showed resistance towards bacterial and fungal growth under the exposure of treatment oils and water.

## 1. Introduction

In the recent years, there has been a growing interest to develop sustainable and eco-friendly materials for a variety of applications using the waste generated from various sources. The agricultural sector produces organic waste from different crops such as cereals, sugarcane, coconut, and live stocks, etc. Rice is one of the majorly harvested crops, and rice husk (RH) and RS are the by-products of the rice crop. In India, 352 Mt of agricultural stubble waste is produced annually by harvesting of rice/paddy, wheat, barley, and maize [1,2]. The majority of these wastes are unutilized and are discarded by burning and dumping in open environment. The RS/RH have intrinsic properties such as a high calorific power (about 4000 kcal/kg), low thermal coefficient, hardness, fibrousness, and abrasive nature. They find applications as building panels for brake pads, thermal and acoustic insulation, etc., [3,4,5,6,7,8,9,10]. The RH is also used as biomass fuel for industrial and domestic heating purposes [11], but it creates a new residue in the form of ash composed of silica (ranging from 80% to 95%). The ash is resistant to chemical etching (acid slag) and thermal shocks (above 600 °C) and has low thermal conductivity and low mechanical properties, making it suitable for producing ceramic material. In addition, it also finds an application as insulating material for cold storage, floors, walls, and roofs. The thermal conductivity of RH ranges from 0.064–0.093 W/M^2^ [12]. Several researchers have explored the potential of RS in polymers for different applications. RS-reinforced epoxy particle boards showed improved tensile strength (*σ_T_*: 0.60 kgf/mm^2^) [13]. Small-sized RS_p_ boards showed higher moisture resistance compared to larger-sized particles. Automotive bumpers developed using RS_p_ showed 16.83% higher *σ_T_* compared to standard bumper materials [14]. A 3-D printed composite using RS powder and poly-lactic acid improved the *σ_T_*-44.66 MPa and flexural strength (*σ_F_*)-76.06 MPa [15]. Reinforcement of RS with polypropylene showed maximum *σ_T_*-7.04 MP and *σ_F_*-40.71 MPa [16]. Results also indicated an improvement in the modulus of the composite due to the addition of RS. The RS-reinforced polyethylene composite showed maximum *σ_T_* (18.2 MPa) and *σ_F_* (24.5 MPa) [17]. Studies showed that the use of agro-wastes improved the wear properties of the polymer composites, which make them suitable for brake pad applications [18,19,20].

The literature shows that the RS can be effectively used for thermal insulation, brake pads, and automotive bumper application. However, the tensile and flexural properties of RS-based polymer composites are low compared to other natural and synthetic fiber-reinforced composites. RS being an organic material finds extensive scope for producing bio/semi-bio composites for structural applications involving lower loads. Recently, health treatments by Ayurveda principles are becoming more attractive, particularly stress management and physiotherapy [21,22,23,24]. Ayurveda provides a wide range of treatment for certain diseases through procedural interventions such as *Shastrapranidana, Bahirparimarjana,* and *Antarparimarjana chikitsa*. The external therapies include simple oil massages and various sudation therapies [25]. Internal purification therapies are combined with external therapies such as massage, steam, etc., which are done on specially designed treatment tables with the following dimensions: length 4 *Hasthas*, 1 *Hastha* wide, and ¼ *Hastha* in height [*Dharakalpa*]. These tables are made using wood of plant species, namely, Plaksha, Udumbara, Gandhasara, Nyagrodha, Devadruma, Punnaga, Kapitha, Chocha, Bakula Ashoka, Asana, Amra, Arjuna, Agnimantha, Amogha, Khadira, Nimba, Bilwa, Champaka, Dola, etc. [26,27]. Although wood is an ideal material for this application in the era of deforestation, the availability of quality wood, its cost, and its maintenance are great challenges [28]. In this view, synthetic materials such as polyvinyl chloride and polyurethane combined with glass fiber fabric are used in the making of therapeutic tables. Such reinforcement may cause fiber glass dermatitis, which includes symptoms such as redness of the affected skin, itching, fissure of skin, etc. The extent to which it may affect a person depends on the duration of exposure, the depth of penetration into the skin, and environmental factors such as humidity, temperature, etc. With the growing consciousness regarding this issue among the public, patients prefer to use organic materials for their treatment. Hence, wood-based equipment or that which contains organic materials as major components is gaining popularity compared to synthetic-based materials. In this view, the present work investigates the mechanical and liquid absorption properties of RS-based composites for Ayurvedic therapy tables. The results obtained from this work are promising from the following viewpoints. The RS_p_ composite exhibits the requisite properties suitable for making eco-friendlier ayurvedic treatment tables. In addition to this, the use of RS provides a sustainable solution for the replacement of synthetic fibers for certain applications. This has direct societal and environmental implications by generating additional revenue to the paddy growers, which directly addresses the solution to problems associated with disposal of agro-waste generated from the harvesting of RS.

## 2. Materials and Methods

### 2.1. Materials

**Figure 1:**
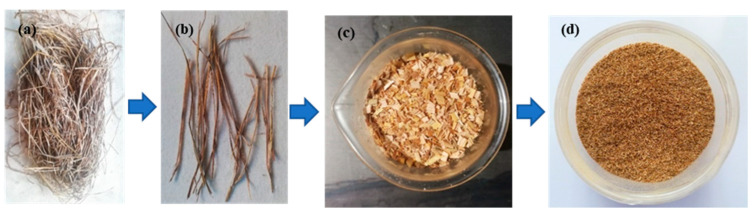
Preparation of RS particles (**a**) Bale, (**b**) Segregation, (**c**) Particles and (**d**) Fine RS particles.

Epoxy resin (Lapox L12) and hardener (K6) were used for the preparation of the test samples and were procured from Atul Pvt. Ltd., Gujarat, India. The pot life of epoxy and hardener mixture is 30–40 min, and the curing time is between 14 h to 24 h at a standard temperature of 25 °C. The properties of the epoxy and hardener material used are shown in Table 1. The RS used for the fabrication was collected from agricultural fields in Udupi, India. The RS was segregated and washed with distilled water to remove the residual content and then dried in an oven at a temperature of 80 °C. The RS was then slit along the straw direction and was chopped into particles with a size less than 2 mm and then ground to produce fine RS_p_. Figure 1 details the stages of RS_p_ preparation.

### 2.2. Preparation of RSp-Reinforced Composite

**Figure 2:**
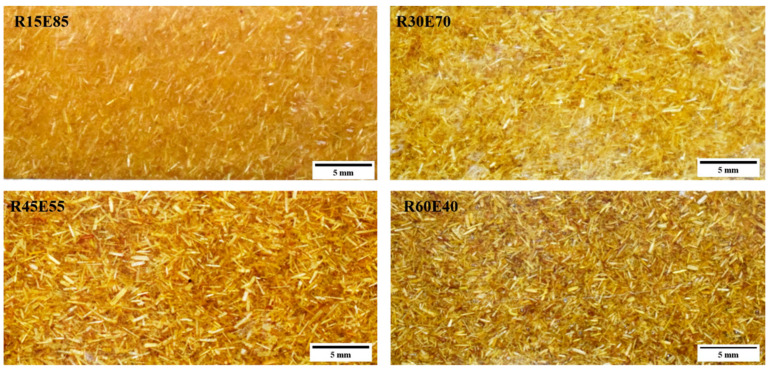
RS_p_-reinforced test samples.

The hand layup method was used to fabricate the RS_p_ test samples [29,30,31]. Table 2 shows details of different test samples prepared by varying the volume fraction (V_F_) of RS_p_. The mold cavity was coated with polyvinyl alcohol-releasing agent to easily remove test samples from the mold. The RS particles were mixed with epoxy to form a uniform matrix mixture, and then the hardener was added at a ratio of 1:10. This homogenous mixture was poured into the mold cavity, and a brush was used to uniformly spread the mixture throughout the mold cavity to achieve a homogeneous structure. A metal plate whose surface was coated with releasing agent was placed on the mold to apply uniform pressure on top of the mold cavity. The laminate was cured at atmospheric temperature for 24 h and post cured at 80 °C for 30 min in a furnace. Figure 2 shows the distribution of RS_p_ in the composite.

### 2.3. Physical and Mechanical Properties

#### 2.3.1. Density and Void Content

The experimental density (*ρ_ce_*) of the test samples was evaluated by Archimedes’ principle. A test sample of known weight was immersed in a flask filled with distilled water, and the water displaced by the test sample was measured. The average *ρ_ce_* of the test sample was calculated based on the result of 10 trials. The theoretical density (*ρ_ct_*) of the RS_p_ composites was calculated based on Equation (1). The void content (*V_c_*) in the test samples was calculated by considering the *ρ_ce_* and *ρ_ce_* of the composite using Equation (2), where *ρ_m,f_* and *V_m,f_* are the density and volume fraction of matrix and fiber material, respectively [32,33].

(1) $$\rho_{ct}=\rho_{m}V_{m}+\rho_{f}V_{f}$$

(2) $$V_{c}=\frac{\rho_{ct}-\rho_{ce}}{\rho_{ct}}\times \;100$$

#### 2.3.2. Microhardness

The microhardness test of the prepared RS_p_ composites was evaluated by a Vickers hardness tester (Make: MMT-X, Matsuzawa Co., Ltd., Toshima, Akita, Japan) [34]. Using a diamond indenter, a load of 100 g was applied to the test sample for a dwell period of 10 s to produce the indentation on the surface of the specimen (50 mm × 25 mm × 3 mm). The diagonal lengths of the indented region on the test sample surface were measured using a microscope. The Vickers hardness number (*V_h_*) was calculated using Equation (3), where *F_a_* is the applied forced and *A_i_* is the indentation area. The measurements were repeated for five trials on different indentation locations on the test sample.

(3) $$V_{h}=1.854\frac{F_{a}}{A_{i}{}^{2}}$$

#### 2.3.3. Tensile Strength of the RS and RS_p_ Composite

**Figure 3:**
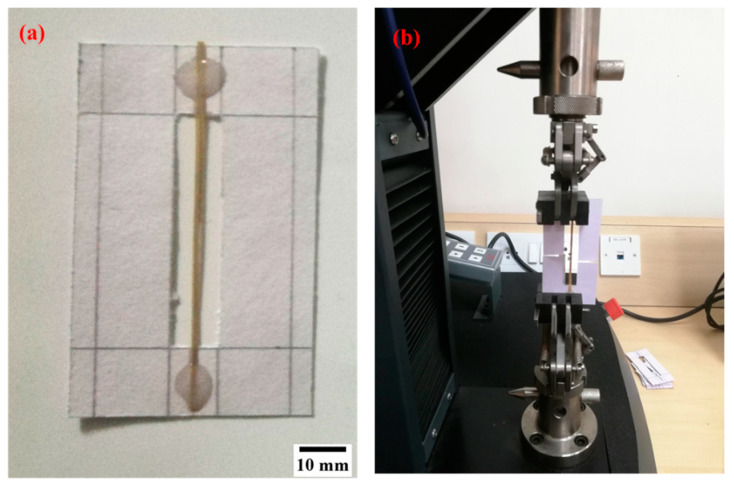
Tensile test of the RS fiber. (**a**) RS test sample; (**b**) Testing instrument.

The *σ_T_* of the RS fiber was evaluated as per the ASTM C1557-20 standard. The RS fibers were split into quarters along the fiber direction to form a single fiber strand. A mounting tab was used to load the RS fiber into the testing machine. The ends of the RS fiber with a fiber gauge length of 50 mm were glued using adhesive (epoxy). Figure 3a shows the mounting tab with the RS fiber. As shown in Figure 3b, the mounting tab was placed in the grips of the testing machine (Make: WANCE ETM-A, Shenzen WANCE testing machine Co., Ltd., Beijing, China), and the sides of the mounting tab were cut to release the support of the cardboard structure without damaging the RS. The test was carried out by maintaining a cross-head speed of 0.2 mm/min until the fiber failed. The *σ_T_* of the RS fiber was calculated using Equation (4), where *F_m_* is the maximum force and *A_f_* is the cross-sectional area of the fiber.

(4) $$\sigma_{T}=\frac{F_{m}}{A_{f}}$$

The *σ_T_* and *σ_F_* of the test samples was evaluated using a universal testing machine (Make: UNITEK 9940, Fuel Instruments and Engineers Pvt. Ltd., Kolhapur, Maharastra, India). The *σ_T_* and *σ_F_* of the test samples was evaluated as per the ASTM D 3039 and ASTM D790 standards, respectively. A cross-head speed of 2 mm/min was maintained during the test. The maximum failure load of the test samples was recorded, and *σ_T_* and *σ_F_* were calculated using Equations (5) and (6), respectively, where *A_c_*, *L*, *w*, and *d* are the cross-sectional area (mm^2^), length (mm), width (mm), and thickness (mm), respectively [35,36,37].

(5) $$\sigma_{T}=\frac{F_{m}}{A_{c}}$$

(6) $$\sigma_{F}=\frac{3F_{m}L}{2wd^{2}}$$

#### 2.3.4. Liquid Absorption and Microbial Study

The liquid absorption rate (*L_a_*) of the test samples was evaluated as per the ASTM D570–98 standards by immersing the tests samples in water and Kottakal Dhanvantaram Thailam oil (KDT). Test samples with 50 mm × 2.5 mm × 3 mm dimensions were prepared for the liquid absorption test. The initial weights of the test samples were recorded and then immersed in normal tap water and KDT oil individually. The weight of each test sample was measured at regular intervals of 24 h after immersion. The water and oil absorption rate of the specimens was calculated by using Equation (7), where *W_1_* and *W_2_* are the initial and final weight (g) of the test samples. Further, the formation and growth of microorganisms in the prepared test samples under the influence of moisture and KDT oil were evaluated by conducting a microbial study. The test samples were continuously immersed in KDT oil and bacterial suspension solution for 15 days, and the attachment of microorganisms on the RS_p_ test samples was studied [38,39].

(7) $$L_{a}=\frac{W_{2}-W_{1}}{W_{2}}\times \;100$$

## 3. Results

### 3.1. Morphological Study of Rice Straw

**Figure 4:**
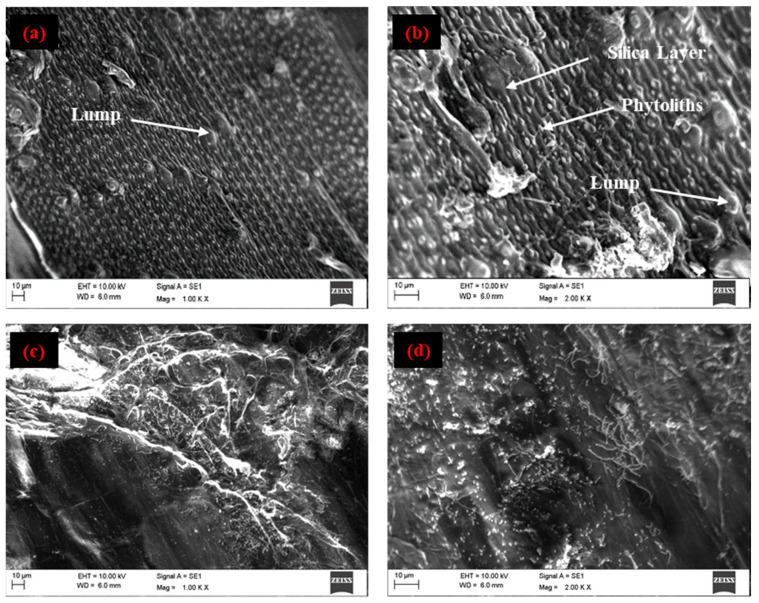
Microscopic view. (**a**,**b**) outer surface and (**c**,**d**) inner surface of RS.

The morphological features of untreated RS were examined using a SEM (Make: EVO MA18, Carl Zeiss Ltd., Cambridge, UK). The structure of the inner and outer surfaces of untreated RS was examined by splitting the RS along its axis. Figure 4a–d shows the SEM images at different locations of the outer and inner surfaces of RS. The outer surface of RS shows large globular and small spiked symmetrically arranged grid-like structures of phytoliths [40,41]. These phytoliths occurred in different shape and size throughout the RS surface [42]. These structures were covered by a silica layer and are responsible for the hydrophobic nature of RS [43,44]. The variation in the surface roughness between the inner and outer surfaces of the RS was clearly visible in the SEM images. The comparison of their images shows that the inner surface of RS appeared to be smoother compared to its outer surface.

### 3.2. Density

**Figure 5:**
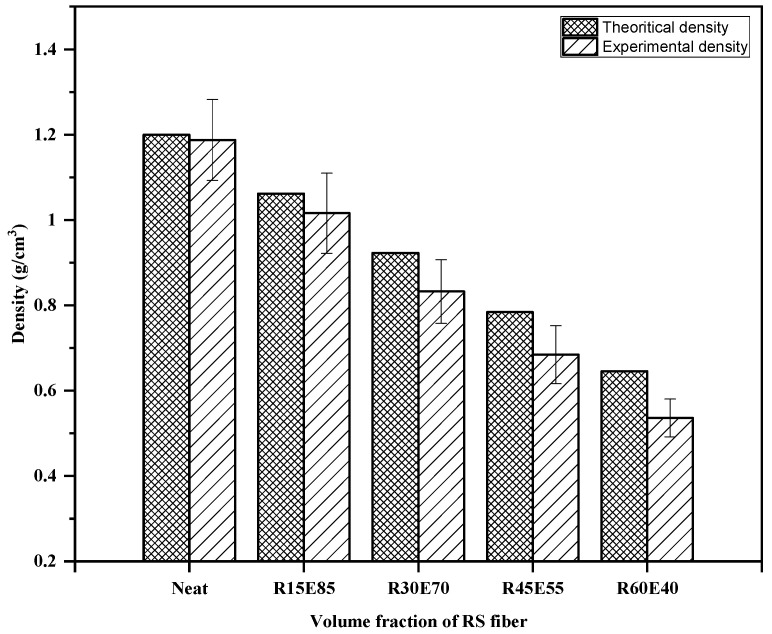
Density of composites.

**Figure 6:**
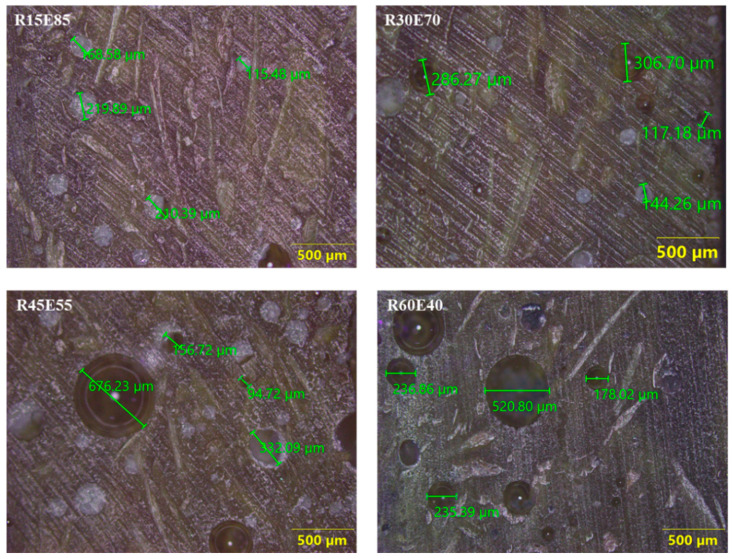
Optical microscope images of voids in the test samples.

The average density of RS_p_ was 0.27 ± 0.013 g/cm^3^. The *ρ_ct_* and *ρ_ce_* of the test samples are shown in Figure 5. It is seen that the density of the test samples decreased with an increase in the RS content in the epoxy. The *ρ_ce_* of R15E85, R30E70, R45E55, and R60E40 composites was reduced by 14.44%, 29.89%, 42.39%, and 54.86%, respectively compared with neat epoxy composite. This reduction in the density of RS_p_ composites is mainly due to the lower density of RS_p_ compared to the epoxy material. It was also observed that the *ρ_ce_* of the test samples was lower than *ρ_ct_*. This is primarily due to the presence of small voids that are formed during the fabrication of test samples. Complete elimination of such voids is unavoidable, and they are generated due to the entrapment of gases generated from the chemical reaction between the epoxy and hardener during the fabrication process [45,46]. It was observed that the void content in test samples increased with increased RS_p_ in epoxy due to the increased agglomerations at higher RS_p_ concentrations. These agglomerated zones prevented the escape of gases and produced voids of varied size (94 µm to 676 µm) in the test sample, as observed in Figure 6.

### 3.3. Microhardness

**Figure 7:**
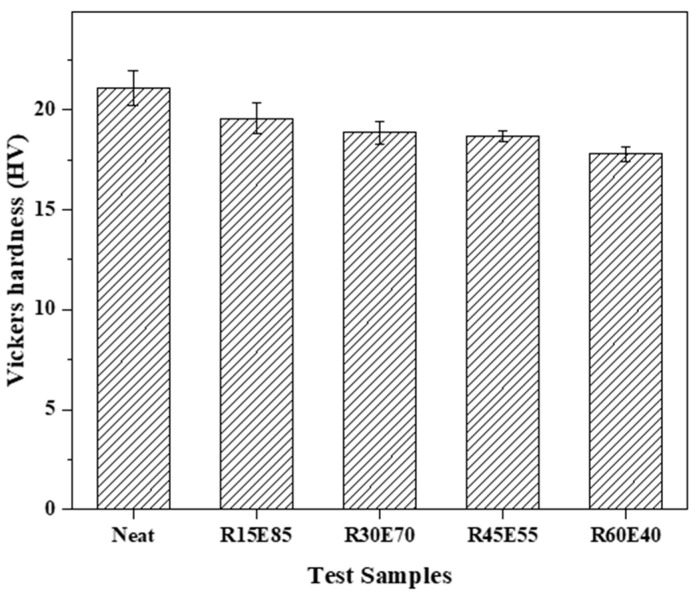
Vickers hardness of test samples.

Figure 7 shows the surface hardness values of the test samples. The neat epoxy had the highest hardness (21.1 HV). It was observed that the surface hardness of the RS_p_ test samples decreased with an increase in the RS_p_ content compared to the hardness of the neat epoxy test sample. This is due to the soft nature of RS_p_ reinforcement embedded in the epoxy matrix. The load applied to the surface of the test samples was effectively absorbed by the soft RS_p_. Such an interaction between the RS_p_ and epoxy material [47] led to a marginal reduction in the hardness of the test samples with a higher RS_p_ content.

### 3.4. Tensile Properties

**Figure 8:**
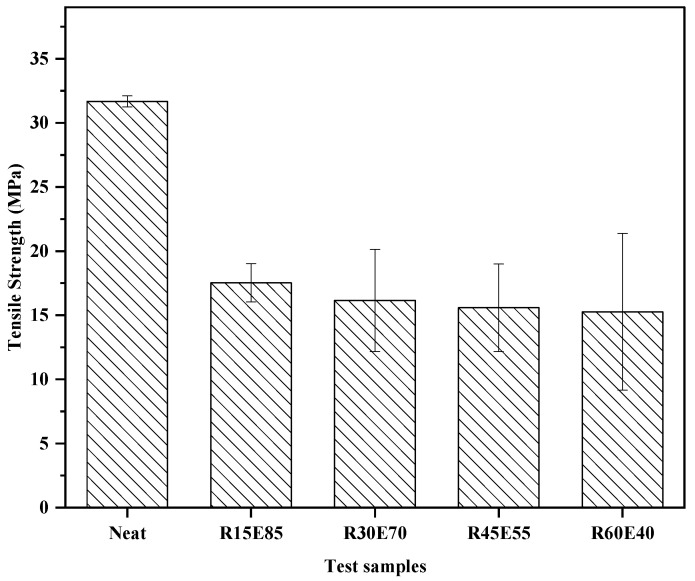
Tensile strength of the test samples.

The average *σ_T_* of RS fiber based on five experimental trials was calculated to be 180.04 MPa. Figure 8 shows the *σ_T_* of the test samples.

The neat epoxy had the highest *σ_T_* (31.67 MPa) compared to R15E85, R30E70, R45E55, and R60E40. The *σ_T_* of the composite decreased with an increase in the RS_p_ content. The *σ_T_* was reduced by 12.89% by increasing the RS_p_ content from V_F_: 0.15 to V_F_: 0.60. This was due to the randomly oriented discontinuous reinforcement phase in the matrix. As discussed earlier, the RS_p_ agglomeration and void content increased with an increase in the RS_p_ content. The combined effect of the above and the weak interfacial bonding between the RS_p_ and epoxy material resulted in the poor transfer of the load from the matrix phase to the reinforcement phase. Hence, the *σ_T_* of the RS_p_ test samples decreased compared to neat epoxy. The higher deviation in the *σ_T_* values of the test samples at a higher RS_p_ content indicated structural inhomogeneity in the test samples. Similar trends were also observed in studies conducted on the tensile properties of natural fiber-reinforced polymer composites [48,49].

**Figure 9:**
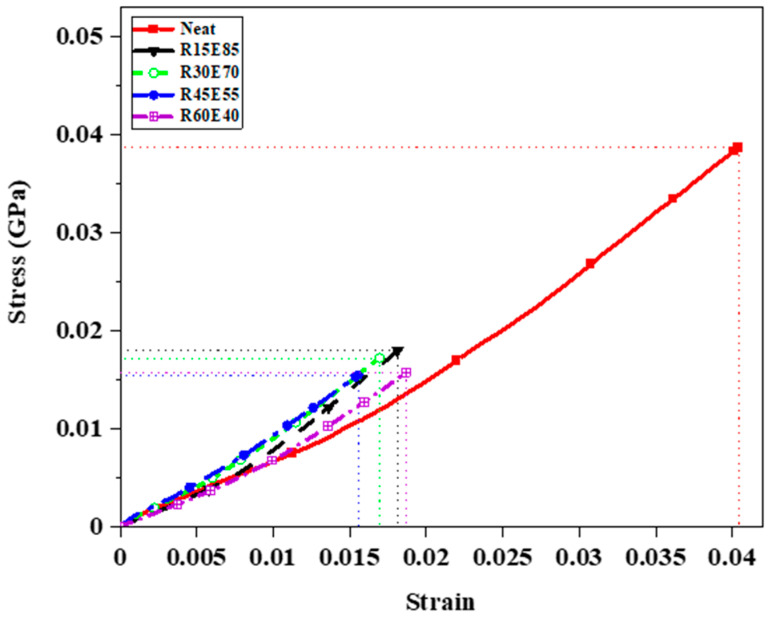
Stress–strain plot of test samples under a tensile load.

Figure 9 shows the stress–strain plot of test samples under a tensile load. The neat epoxy composite had higher strength compared to R15E85, R30E70, R45E55, and R60E40. The strain rate was higher for the neat epoxy composite. The strength of the composites was found to vary marginally with an increase in the RS_p_ content. It is well known that the load applied is transferred to the RS_p_, and the corresponding location acts as a stress-concentration region. There was a non-uniform stress distribution in the test samples, and the maximum stresses were observed near the reinforcement. Due to the applied load, the epoxy matrix near the filler interface broke due to higher stresses, leading to a decrease in the tensile strength of the RS_p_ composite materials. The non-uniformity in the distribution of the stress-concentrated location will increase with an increase in the RS_p_ concentration. Hence, the tensile strength of the composite decreased with an increase in the RS_p_ content. It is also seen from the figure that there was a gain of strain with the increase in the RS_p_ content (0.015–0.02).

### 3.5. Flexural Properties

**Figure 10:**
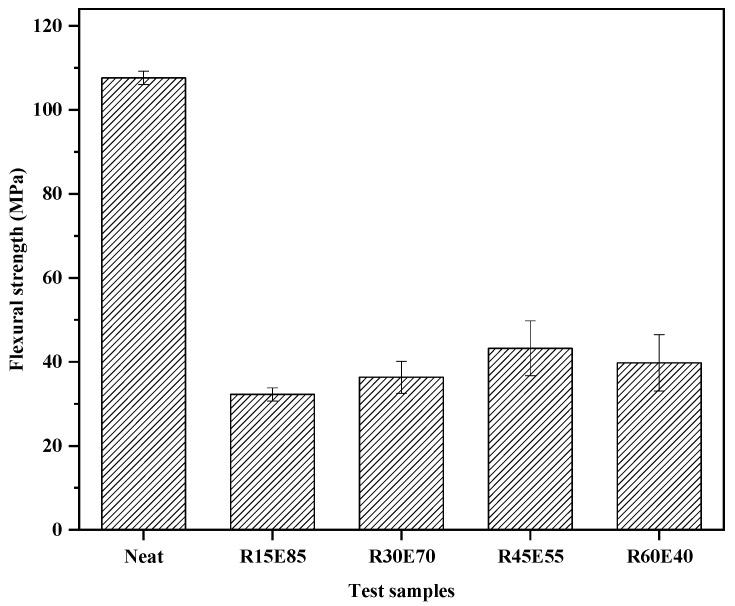
Flexural strength of test samples.

Figure 10 shows the *σ_F_* of the test specimens. The neat composite showed a maximum *σ_F_* (107.6 MPa) compared to the RS_p_ composites. The *σ_F_* increased with an increase in RS_p_ up to RS_p_ V_F_: 0.45, and thereafter, a decreasing trend was observed. The load applied during the flexural test acted in the direction perpendicular to the RS_p_ surface. It is inferred from the results that the applied load was completely absorbed up to a critical volume of RS_p_ (V_F_: 0.45). Loading beyond this concentration resulted in decreasing trend of *σ_F_*. This was attributed due to the formation of voids and agglomerations particularly at higher particle concentrations, which affects the structural integrity of the material [50,51].

**Figure 11:**
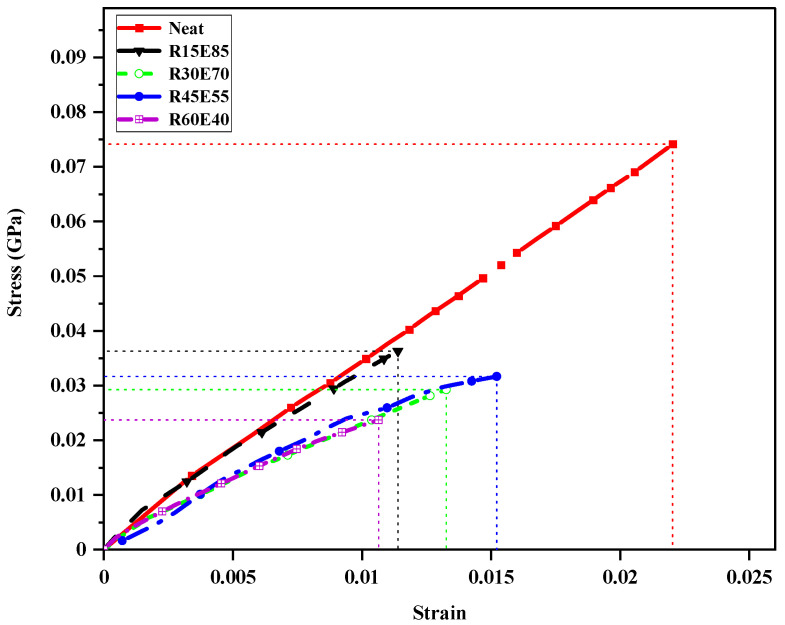
Stress–strain plot of test samples under a flexural load.

Figure 11 shows the stress–strain plot of test samples under a flexural load. The neat composite showed higher stress and strain compared to the RS_p_ test samples. A higher concentration of RS_p_ in epoxy was observed to reduce the strength of the RS_p_ test samples. The higher reinforcement in epoxy resulted in poor wettability of RS_p_ and thus reduced the internal bond strength of the composite. The discontinuous phase of RS_p_ failed to completely absorb the stress generated during the test. It was observed from the figure that higher RS_p_ in epoxy increased the strain in the RS_p_ test samples. A maximum strain of 0.015 was observed for the R60E40 test sample amongst the RS_p_-reinforced test samples.

### 3.6. Fractural Analysis of Tensile and Flexural Test-Failed Specimens

**Figure 12:**
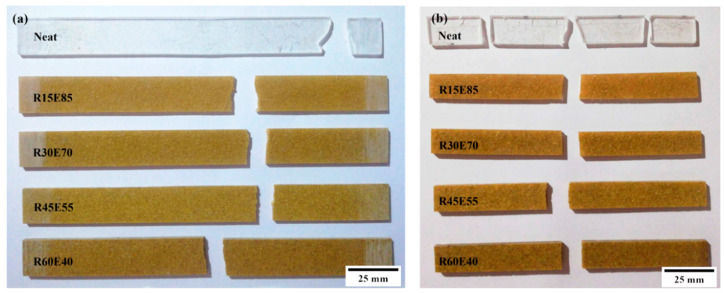
Fractured test samples subjected to (**a**) tensile (**b**) flexural test.

**Figure 13:**
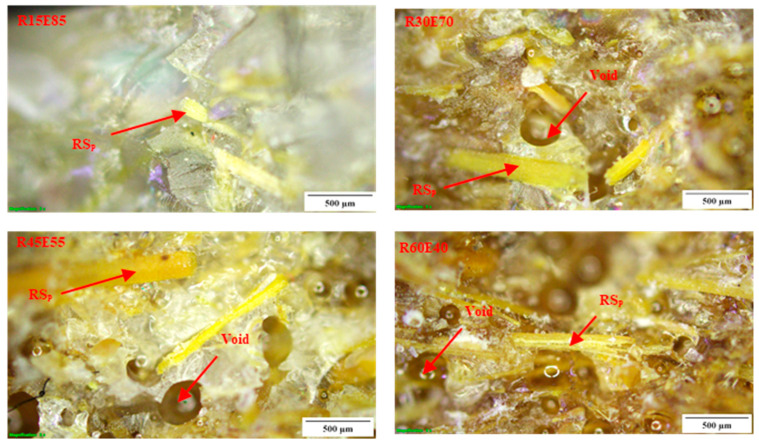
Tensile load-fractured surfaces of test samples.

**Figure 14:**
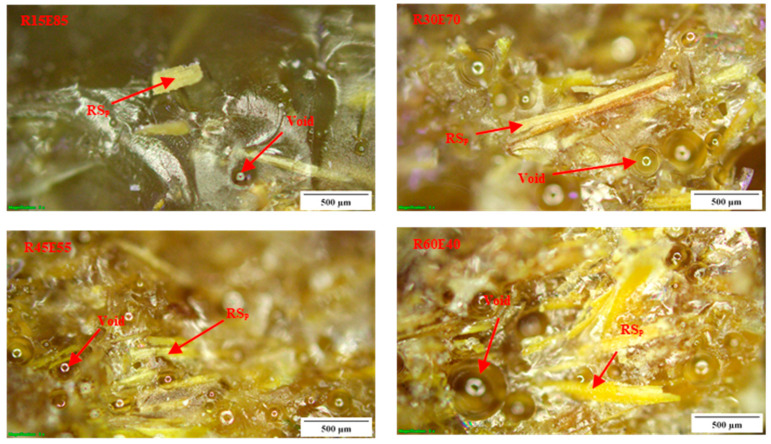
Flexural load-fractured surfaces.

Figure 12a,b shows the fractured surfaces of tensile and flexural test samples, respectively. It is seen that due to the tensile load, the specimens were split at the center of the gauge length. The microscopic observation shows that there were no delamination-related damages on the gripping tab region as well as on other surfaces. The failure of the material occurred at the center of the gauge length, and the fracture line was aligned almost horizontally across the specimens. Figure 13 and Figure 14 show the optical images of the fractured surfaces of test samples subjected to tensile and flexural tests, respectively. The fractured surfaces show that there were no fiber pull-outs from the matrix and damages near the tab section, which indicated the good structural integrity between the RS_p_ and matrix material. Fractured surfaces of test samples showed the presence of voids formed by the entrapment of gases during the curing process.

### 3.7. Liquid Absorption Study

**Figure 15:**
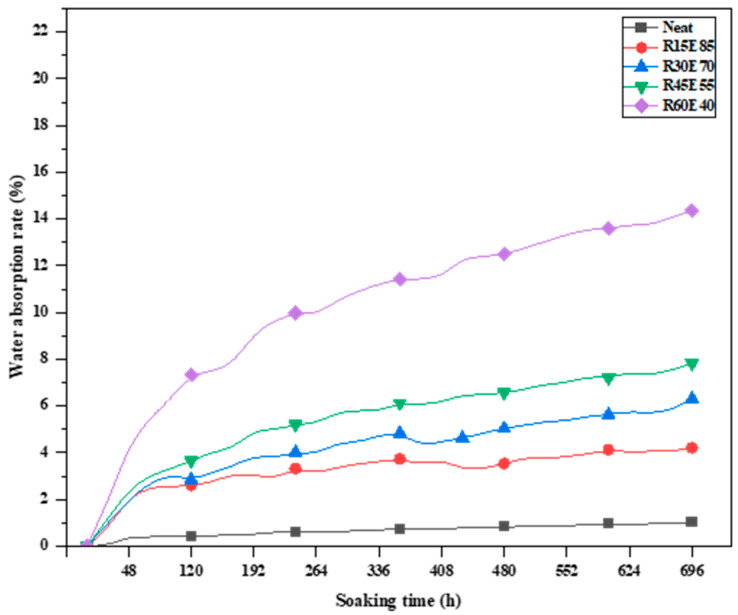
Water absorption rate of the RS_p_ test samples.

The test samples were subjected to the WA test to investigate their water-resistant properties. The test samples were immersed in normal water for 696 h under standard laboratory conditions. Figure 15 shows the WA rate of different test samples. It was observed that the neat epoxy showed almost negligible WA (<0.05%) during the soaking period. Although epoxy material is water-resistant, a very small amount of water was absorbed by the surface voids present in the neat epoxy test sample. Further, it was observed that there was a considerable amount of WA by the test samples with RS reinforcement. The WA rate appeared to increase with higher RS content, and up to RS V_F_: 0.45, the WA rate was less than 7.8%, whereas the R60E40 composite showed the highest WA rate of 14.36%. In the initial 72 h of soaking, the WA rate for all the test samples rapidly increased linearly, and thereafter, the absorption rate decreased and showed an almost constant trend. This was due to the exposure of the RS surface, which is hydrophilic in nature, and the voids present in the surface of the test sample also influenced the moisture uptake [52,53]. After the test sample completely absorbed the water content due to continuous soaking, a saturation point for WA was reached, beyond which the WA rate is marginal [32,54].

**Figure 16:**
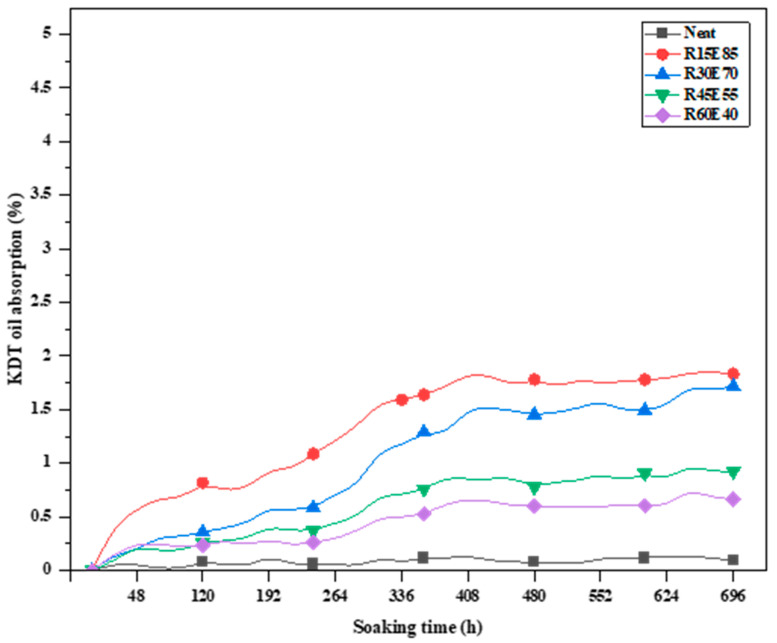
KDT oil absorption rate of the RS_p_ test samples.

All the test samples were subjected to an absorption study by immersing the test samples in KDT oil for 696 h under standard laboratory conditions. Figure 16 shows the KDT oil absorption rate of different test samples measured at an interval of 24 h. The test samples initially showed a linearly increasing trend of oil absorption up to 48 h of the immersion period. As the immersion period increased, the rate of oil absorption gradually stabilized and reached almost a constant value. The test samples were found to completely absorb the KDT oil and reach a saturation point beyond which the increase in the oil absorption rate of the test samples was minimum. The oil absorption rate of the test samples was less than 2%, indicating higher resistance to oil absorption. Comparative analysis of the absorption of water and KDT oil showed that the test samples were more resistant towards oil absorption than water. This is due to the higher viscosity of water compared KDT oil.

### 3.8. Microbial Study of RS_p_ Test Samples

The test samples were investigated for two different types of bacteria, i.e., Gram-negative *Escherichia coli* (ATCC 25922), *Pseudomonas aeruginosa* (ATCC 27853) and Gram-positive cocci *Staphylococcus epidermidis* (clinical strain). The *Candida albicans* (ATCC 90028) fungus was used to study the resistance of the material to fungal growth.

**Figure 17:**
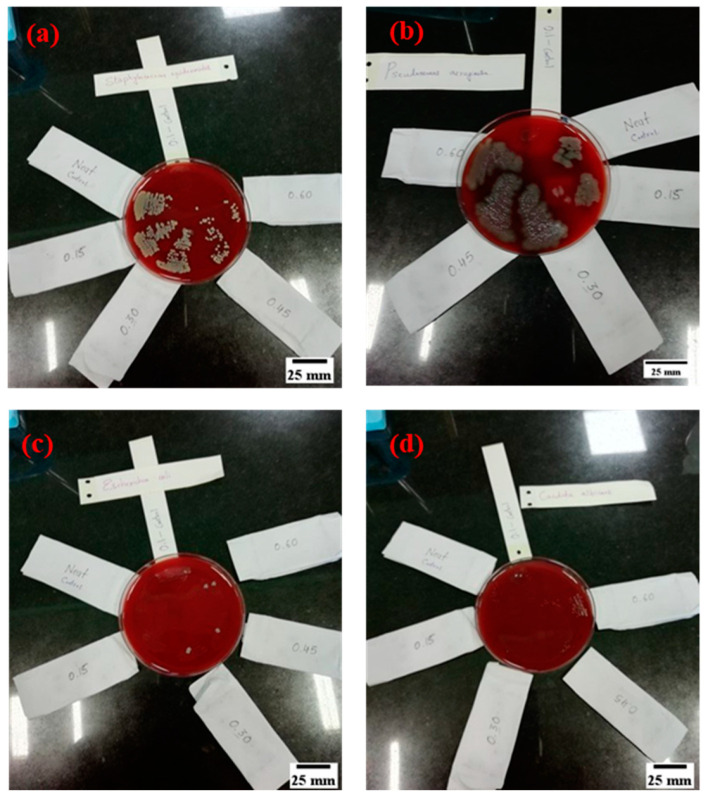
Test samples immersed in bacterial suspension with (**a**) *Staphylococcus epidermidis* (**b**) *Pseudomonas aeruginosa* (**c**) *Escherichia coli* (**d**) *Candida albicans*.

The bacteria from the frozen stock culture were transferred to peptone water supplemented with 5% sheep blood plates and were incubated at 37 °C for 24 h. The bacteria were then transferred to 50 mL of sterile peptone water and grown at 37 °C. The number of bacteria in the bacterial suspension was adjusted to 0.5 MacFarland standard, which is equivalent to 1 × 10^8^ bacteria/mL. Before conducting the bacterial attachment on test samples (50 mm × 25 mm × 3 mm), they were cleaned by immersion in ethyl alcohol solution for 1 h to remove contaminants and microorganisms. Finally, test samples were washed with distilled water [55]. The immersion inoculation method was followed for the bacterial attachment and to grow the bacteria directly on the surface of the test samples [56]. The test samples were placed in a sterile container and completely covered using 20 mL of the bacterial suspension for 1 h at room temperature without shaking. The test samples were continuously immersed in the bacterial suspension and KDT oil mixture for 15 days in order evaluate the growth of bacteria on test samples as shown in Figure 17.

**Figure 18:**
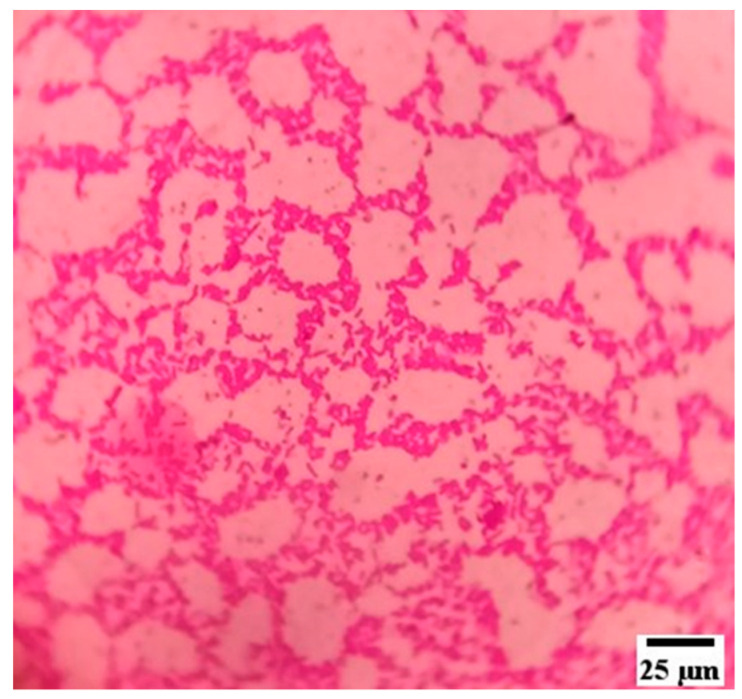
Microscopic image of the bacterial solution (Mag: 1000×).

Finally, the test samples were removed from the bacterial suspension and were washed three times with 20 mL of distilled water for 10 s and allowed to dry under ambient conditions for 1 h before being sampled. Sampling was done using a swab inoculation method with the sterile swab rubbing on the test samples followed by inoculation in 5% sheep blood agar [57]. The growth of the colonies was noted. Controls used were plane glass block and KDT oil. Swabs were also collected from the surface of the bed and the sides of the bed, which was used by the patients for quality control. Figure 18 shows the microscopic image of the bacterial solution. The microbial study showed that the test samples were resistant against the growth of *E. coli* and *Candida albicans* bacteria, whereas *Pseudomonas aeruginosa* and *Staphylococcal epidermidis* growth was observed in the test samples. The continuous contact of KDT oil did not show microbial growth on sheep blood agar. Further, the swabs collected from the patient’s bed did not influence the growth of any microorganisms on the sheep blood agar, indicating that the RS_p_ test samples were resistant against bacterial and fungal growth.

## 4. Conclusions

In the present work, RS_p_-reinforced epoxy composite were investigated for their mechanical and liquid absorption properties to determine the effect of RS_p_ reinforcement. The scope of the present investigation is limited to the material characterization of the mechanical and liquid absorption properties of the RS/Epoxy composite developed using untreated rice straw. There is well-established evidence which shows that the tensile and compressive properties of the untreated fibers are enhanced by the chemical treatment of the fiber. Further, the chemical treatment affects the hydrophilic nature of the fibers, which reduces the moisture absorption of the composite materials. The chemical treatment of the rice straw reduces the straw size, and the process incurs a cost. With the aim of developing cost-effective materials with fewer processing stages for using rice straw after harvesting, the present work is aimed at characterization of the properties of untreated RS/Epoxy composite. The use of RSp reinforcement considerably reduce the quantity of polymer used in making the ayurvedic treatment table and hence provides an eco-friendly solution for this application. Based on the experimental results, the following conclusions are drawn.

- The composites with RS_p_ volume of 15% and 45% had the highest *σ_T_* (17.53 MPa) and *σ_T_* (43.24 MPa), respectively. The addition of RS_p_ at 60% reduced the density by 54.59%, hardness by 15.63%, tensile strength by 51.78%, and flexural strength by 63.04% compared to properties of the neat epoxy material.
- The tensile and flexural test failed specimens showed brittle fracture behavior, and the failures were observed to occur prominently near the locations of voids. Interestingly, fiber pull-outs were not found on the fractured surface. The fiber was split at the location of the crack, and the split fibers were intact with the matrix.
- The RS_p_ composite was resistant to the exposure of water and KDT oil. Soaking of test materials for up to 696 h in KDT oil showed <2% absorption. In addition, the test samples also showed the absence of microbial growth under the exposure of water and KDT treatment oil, indicating the suitability of the material for making ayurvedic treatment tables and accessories.

## Figures and Tables

**Table 1:** Properties of epoxy (As specified by the supplier).

Properties	Range
Density of epoxy (L-12) at 25 °C	1.1–1.2 g/cm^3^
Density of hardener (K-6) at 25 °C	0.95–1.1 g/cm^3^
Tensile strength	55–70 MPa
Flexural strength	120–140 MPa
Impact strength	17–20 KJ/m^2^
Thermal conductivity	0.211 kCal/m h °C
Co-efficient of thermal expansion	64–68 × 10^−6^/°C
Water absorption (25 °C/24 h)	0.5 *w/w* % (Max)

**Table 2:** Composition and coding of test samples.

Sl. No	Sample Code	RS_p_ (V_f_)	Epoxy (V_m_)
1	Neat	0	1
2	R15E85	0.15	0.85
3	R30E70	0.30	0.70
4	R45E55	0.45	0.55
5	R60E40	0.60	0.40

## Data Availability

Data sharing is not applicable.
